# Genome-wide Identification of *PP2C* Genes and Their Expression Profiling in Response to Drought and Cold Stresses in *Medicago truncatula*

**DOI:** 10.1038/s41598-018-29627-9

**Published:** 2018-08-27

**Authors:** Qi Yang, Kun Liu, Xiaocui Niu, Qi Wang, Yongqing Wan, Feiyun Yang, Guojing Li, Yufen Wang, Ruigang Wang

**Affiliations:** 1 Inner Mongolia Key Laboratory of Plant Stress Physiology and Molecular Biology, College of Life Sciences, Inner Mongolia Agricultural University, Hohhot, P. R. China; 2 Key Laboratory of Forage and Endemic Crop Biotechnology, Ministry of Education, School of Life Sciences, Inner Mongolia University, Hohhot, P. R. China; 3 Institute of Microbiology, Chinese Academy of Sciences, Beijing, P. R. China

## Abstract

Type 2 C protein phosphatases (PP2Cs) represent the major group of protein phosphatases in plants and play important roles in various plant processes. In this study, 94 *MtPP2C* genes were identified from *Medicago truncatula* and further phylogenetically classified into 13 subfamilies, as supported by exon-intron organization and conserved motif composition. Collinearity analysis indicated that segmental duplication events played a crucial role in the expansion of *MtPP2C* gene families in *M*. *truncatula*. Furthermore, the expression profiles of *MtPP2Cs* under different abiotic treatments were analyzed using qRT-PCR. Results showed that these *MtPP2Cs* genes displayed different expression patterns in response to drought, cold and ABA stress conditions and some of the key stress responsive *MtPP2Cs* genes have been identified. Our study presents a comprehensive overview of the *PP2C* gene family in *M*. *truncatula*, which will be useful for further functional characterization of MtPP2Cs in plant drought and cold stress responses.

## Introduction

Reversible phosphorylation of proteins is an important protein modification process that regulates a large number of physiological and biochemical reactions in plants. Phosphorylation and dephosphorylation are catalyzed by protein kinases (PKs) and protein phosphatases (PPs), respectively. According to the specificity of substrates, PPs are divided into serine/threonine protein phosphatase (PSPs) and tyrosine protein phosphatases (PTPs). PSPs are classified into two categories: Category 1 includes PP1, PP2A, PP2B, PP4, PP5, and PP6; while category 2 is PPM (protein phosphatase M), including PP2C and other Mg^2+^-dependent phosphatases^1,2^.

PP2C proteins belong to monomer enzymes and the activity depends on Mg^2+^ and Mn^2+^. In eukaryotes, the catalytic domain of PP2C proteins is located at either the N-terminus or the C-terminus^3^. Further research revealed that the regions of catalytic domain in eukaryotic PP2C proteins are relatively conserved, whereas the regions of non-catalytic domain have diverse amino acid sequences^2,3^.

PP2Cs are evolutionarily conserved from prokaryotes to higher eukaryotes, having been found in archaea, bacteria, fungi, plants and animals^4^. In plants, PP2Cs form the largest family of phosphatase genes, accounting for 60–65% of all phosphorylases^5,6^. The high proportion of *PP2C* genes is indicative of their evolutionary significance, requirement and involvement in diverse plant cellular functions^2^. As a major class of protein phosphatases, PP2Cs catalyze dephosphorylation of substrate proteins to regulate signaling pathways and participate in various physiological and biochemical processes in plants. Current studies have shown that PP2Cs play crucial roles in different processes, such as ABA signaling, biotic and abiotic stress responses, plant immunity, K^+^ nutrient signaling and plant development^2,7^.

Drought, salt, and temperature stresses are major environmental factors that affect the geographical distribution of plants in nature, limit plant productivity in agriculture, and threaten food security^8^. Plants evolve a variety of signaling mechanisms to adapt to adverse environments, such as drought, high salt, extreme temperatures and pest attacks. Many studies have shown that some *PP2C* genes are involved in the regulation of the ABA signaling pathway by modulating the kinase activity of SnRK or MAPK to respond to abiotic stresses^9^. For example, PpABI1A and PpABI1B, the only two subfamily A PP2Cs in moss, are directly involved in ABA responses, including induced vegetative desiccation tolerance^10^. In higher plants, the function of PP2C in abiotic stress is more diverse. For instance, in Arabidopsis, ABI1, ABI2 and HAB1 participate in plant abiotic stress/tolerance by negatively regulating ABA signaling^11–13^. Transgenic studies in maize and Arabidopsis confirmed that ZmPP2C-A10 functions as a negative regulator of drought tolerance as well^9^. Similar results have been obtained from studies in other plants, such as tomato^14^, poplar^15^, *Artemisia annua* L^16^., *Populus euphratica*^17^, and sweet cherry^18^. These studies show that subfamily A PP2Cs in Arabidopsis and other plants negatively regulate ABA and stress signaling pathways. On the contrary, BdPP2CA6, a subfamily A PP2C from *Brachypodium distachyon*, was found to be a positive regulator in both ABA and stress signaling pathways^19^. Another study has identified a potential rice subfamily A PP2C, which regulates ABA signaling negatively and abiotic stress signaling positively^20^. Most subfamily A PP2C members of Arabidopsis participate in stress tolerance via ABA-dependent signaling pathways, but in other studies, some PP2Cs can also regulate plant stress tolerance by ABA-independent signaling pathways, such as OsPPOs from rice^21^. These studies indicate that PP2Cs in different plants have diverse functions in stress signal pathways.

As genome sequencing of more species is completed, the *PP2C* gene family has been isolated, identified, and characterized in a number of plant species including Arabidopsis^3,22,23^, rice^6,22^, hot pepper^24^, wild soybean^25^, maize^26^ and *Brachypodium distachyon*^4^. However, as a model legume plant, the *PP2C* gene family in *M*. *truncatula* has not been analyzed comprehensively and their functions remain elusive at present.

In this study, we identified 94 *MtPP2C* genes from *M*. *truncatula* genome and grouped them into 13 subfamilies. Comprehensive analyses of gene structures, gene duplications, chromosomal distribution, and phylogeny of these *MtPP2Cs* were further carried out. At the same time, their expression profiles were also investigated by qRT-PCR under drought and cold treatments. The results presented here provide a solid foundation for further functional characterization of *MtPP2C* genes in this model species.

## Result

### Genome-wide Identification of *PP2C* Family Members in *M*. *truncatula*

To identify the *PP2C* genes, we searched the *M*. *truncatula* genome database (Plaza3.0 database) using the InterPro PP2C domain “IPR001932” as the key word and found 95 putative *PP2C* genes. After confirming the presence of PP2C domains using Pfam and Batch CD-search, we found that one putative *PP2C* gene lacks the PP2C catalytic domain. Therefore, 94 genes were identified as PP2C members in *M*. *truncatula* and were named as MtPP2C1 to MtPP2C94, based on their locus ID.

All of the basic information on these 94 *MtPP2C* genes is provided in Table 1. Sequence analysis revealed that the lengths of the deduced MtPP2C proteins vary from 118 amino acids (MtPP2C71) to 1,256 amino acids (MtPP2C23), with an average of 419 amino acids. The predicted molecular weights (MW) and isoelectric points (pI) range from 13.047 kDa (MtPP2C71) to 133.232 kDa (MtPP2C23) and from 3.80 (MtPP2C23) to 9.82 (MtPP2C84), respectively. Subcellular localization prediction showed that most of the MtPP2C proteins might be located in chloroplasts, nuclei or cytoplasm, followed by mitochondria, extracellular compartments and vacuoles (Table 1).

**Table 1:** List of identified *PP2C* genes in *M*. *truncatula* with their detailed information and localization.

Locus ID	Gene name	Size (aa)	Mass (Da)	pI	Subcellular localization	Chromosome location
Medtr1g013400	*MtPP2C1*	337	36552.76	5.02	vacu	chr1:3433635..3439406 reverse
Medtr1g014190	*MtPP2C2*	396	43773.30	5.08	extr	chr1:3077750..3082294 forward
Medtr1g014640	*MtPP2C3*	337	36552.76	5.02	vacu	chr1:3407349..3413810 forward
Medtr1g015110	*MtPP2C4*	553	59927.62	4.59	chlo	chr1:3710663..3716307 reverse
Medtr1g016620	*MtPP2C5*	344	37956.09	5.62	cyto	chr1:4473372..4477359 forward
Medtr1g019760	*MtPP2C6*	428	46615.23	5.35	vacu	chr1:6004898..6010964 reverse
Medtr1g022030	*MtPP2C7*	508	54464.98	6.83	cyto	chr1:6777081..6782301 reverse
Medtr1g028300	*MtPP2C8*	396	42990.51	6.54	mito	chr1:9502076..9505086 forward
Medtr1g041475	*MtPP2C9*	274	31191.74	8.83	chlo	chr1:15564924..15567717 reverse
Medtr1g050520	*MtPP2C10*	654	72818.36	6.28	chlo	chr1:19689741..19697531 reverse
Medtr1g067210	*MtPP2C11*	375	41560.88	6.90	nucl	chr1:28937740..28940160 reverse
Medtr1g071370	*MtPP2C12*	1071	121605.42	5.88	vacu	chr1:31666298..31681379 forward
Medtr1g075730	*MtPP2C13*	278	32484.43	9.48	mito	chr1:33552419..33554806 forward
Medtr1g083690	*MtPP2C14*	352	39057.09	5.72	nucl	chr1:37239946..37243765 forward
Medtr1g083750	*MtPP2C15*	423	45503.11	7.53	chlo	chr1:37276915..37280425 reverse
Medtr1g085530	*MtPP2C16*	892	99368.06	5.86	nucl	chr1:38194845..38201639 reverse
Medtr1g086350	*MtPP2C17*	390	43078.48	4.81	Nucl,cyto	chr1:38640292..38643291 reverse
Medtr1g106855	*MtPP2C18*	379	42381.22	6.32	chlo	chr1:48356084..48359019 forward
Medtr1g110210	*MtPP2C19*	327	35527.82	8.12	cyto	chr1:49705150..49706843 forward
Medtr1g112840	*MtPP2C20*	397	43841.86	8.18	chlo	chr1:51132582..51136407 forward
Medtr1g115570	*MtPP2C21*	347	37648.21	5.10	cyto	chr1:52256072..52260436 forward
Medtr1g116260	*MtPP2C22*	379	41987.94	8.08	mito	chr1:52552351..52555813 forward
Medtr2g008590	*MtPP2C23*	1256	133232.52	3.80	chlo	chr2:1541912..1549840 reverse
Medtr2g008850	*MtPP2C24*	281	30779.91	6.00	nucl	chr2:1663259..1666800 forward
Medtr2g020970	*MtPP2C25*	439	47485.12	5.13	chlo	chr2:7064747..7067687 reverse
Medtr2g033000	*MtPP2C26*	368	40549.97	4.86	nucl	chr2:12442268..12444743 forward
Medtr2g033910	*MtPP2C27*	373	41406.27	7.76	chlo, cyto	chr2:12929002..12934577 reverse
Medtr2g040500	*MtPP2C28*	545	60033.03	4.80	nucl	chr2:17771507..17774251 forward
Medtr2g078760	*MtPP2C29*	333	36857.77	7.82	chlo	chr2:32964994..32968595 forward
Medtr2g090190	*MtPP2C30*	470	51809.99	5.01	nucl	chr2:38268714..38271536 reverse
Medtr2g093685	*MtPP2C31*	219	23980.67	8.21	extr	chr2:39942441..39943100 reverse
Medtr2g435550	*MtPP2C32*	385	43439.06	6.72	chlo	chr2:13732574..13737504 reverse
Medtr3g031360	*MtPP2C33*	309	33670.96	5.12	chlo	chr3:26807600..26811666 forward
Medtr3g032590	*MtPP2C34*	438	49453.31	9.27	chlo	chr3:10298146..10299844 forward
Medtr3g032660	*MtPP2C35*	432	48461.45	6.88	nucl	chr3:10318461..10320276 forward
Medtr3g032700	*MtPP2C36*	432	48503.53	7.21	nucl	chr3:10335705..10337392 forward
Medtr3g068200	*MtPP2C37*	388	42920.03	5.32	nucl	chr3:30835692..30837501 forward
Medtr3g074610	*MtPP2C38*	282	31073.13	7.75	chlo	chr3:33724920..33727721 forward
Medtr3g091060	*MtPP2C39*	364	40395.37	6.34	chlo	chr3:41371122..41377528 reverse
Medtr3g101540	*MtPP2C40*	429	46753.33	5.77	vacu	chr3:46733738..46738711 forward
Medtr3g104710	*MtPP2C41*	549	59510.52	4.79	chlo	chr3:48269729..48274062 forward
Medtr3g105730	*MtPP2C42*	299	32301.73	5.11	cyto	chr3:48767036..48770816 reverse
Medtr3g105880	*MtPP2C43*	362	39957.92	5.12	chlo,nucl	chr3:48831424..48836500 forward
Medtr3g107880	*MtPP2C44*	381	41655.12	5.96	nucl	chr3:49775982..49778302 reverse
Medtr3g451410	*MtPP2C45*	177	19629.19	9.00	chlo	chr3:18557087..18557752 forward
Medtr3g464650	*MtPP2C46*	318	34776.24	6.02	chlo	chr3:26001759..26003904 forward
Medtr3g464700	*MtPP2C47*	334	36410.85	6.08	chlo	chr3:26016801..26018299 forward
Medtr3g491830	*MtPP2C48*	390	43503.68	7.06	chlo	chr3:41806436..41810585 forward
Medtr4g007440	*MtPP2C49*	364	40021.16	5.23	nucl	chr4:1091195..1100704 reverse
Medtr4g013295	*MtPP2C50*	357	39237.48	6.01	extr	chr4:3704225..3706827 forward
Medtr4g037470	*MtPP2C51*	479	53223.74	5.11	chlo	chr4:14958614..14963747 forward
Medtr4g063905	*MtPP2C52*	704	78658.40	5.44	chlo	chr4:23796219..23800131 reverse
Medtr4g076560	*MtPP2C53*	491	54609.98	5.99	chlo	chr4:29279043..29281997 forward
Medtr4g094208	*MtPP2C54*	278	30311.06	6.71	nucl	chr4:37425469..37430693 reverse
Medtr4g094542	*MtPP2C55*	364	40340.89	4.90	chlo	chr4:38196224..38198698 forward
Medtr4g098650	*MtPP2C56*	779	85337.58	5.43	nucl	chr4:40668226..40674476 forward
Medtr4g113210	*MtPP2C57*	257	29091.05	7.07	cysk	chr4:46533626..46535392 reverse
Medtr4g113345	*MtPP2C58*	341	39320.28	8.91	cyto	chr4:46588605..46592821 reverse
Medtr4g113480	*MtPP2C59*	554	62973.99	5.61	cyto	chr4:46648593..46652101 forward
Medtr4g116420	*MtPP2C60*	384	42990.49	4.89	nucl	chr4:48228380..48231422 forward
Medtr4g118340	*MtPP2C61*	399	44042.09	5.43	chlo	chr4:49023234..49028186 forward
Medtr4g119830	*MtPP2C62*	500	54688.56	5.02	nucl	chr4:49656992..49659929 reverse
Medtr4g120410	*MtPP2C63*	362	40623.83	8.81	cyto	chr4:49916027..49918669 forward
Medtr4g123080	*MtPP2C64*	381	42258.97	5.20	nucl	chr4:50809344..50811831 reverse
Medtr4g125810	*MtPP2C65*	513	56572.79	5.05	chlo	chr4:52217353..52220723 reverse
Medtr5g005810	*MtPP2C66*	583	65595.22	5.66	chlo	chr5:629189..635487 forward
Medtr5g009370	*MtPP2C67*	334	36584.56	5.96	cyto	chr5:2222866..2224264 forward
Medtr5g019790	*MtPP2C68*	450	48234.48	8.32	chlo	chr5:7501168..7504361 reverse
Medtr5g024340	*MtPP2C69*	379	41209.84	5.71	cyto	chr5:9794141..9796708 reverse
Medtr5g063940	*MtPP2C70*	282	30692.76	8.26	chlo	chr5:26529712..26533899 forward
Medtr5g065180	*MtPP2C71*	118	13047.33	8.86	nucl	chr5:27391180..27392171 forward
Medtr5g071550	*MtPP2C72*	378	41099.37	7.05	chlo	chr5:30372747..30375121 forward
Medtr5g080680	*MtPP2C73*	391	42953.52	4.98	nucl	chr5:34535790..34538105 forward
Medtr6g081850	*MtPP2C74*	321	35296.14	8.19	cyto	chr6:30528814..30534664 reverse
Medtr6g087000	*MtPP2C75*	1072	119581.77	4.94	nucl,cyto	chr6:33528353..33537341 reverse
Medtr7g021530	*MtPP2C76*	452	49512.02	5.40	nucl	chr76831281..6837332 reverse
Medtr7g025640	*MtPP2C77*	202	22598.21	6.64	chlo	chr7:8548988..8549923 forward
Medtr7g029240	*MtPP2C78*	318	46612.52	8.56	chlo	chr7:10320773..10322053 forward
Medtr7g060770	*MtPP2C79*	555	61760.66	5.77	chlo	chr7:21968314..21971174 reverse
Medtr7g070510	*MtPP2C80*	447	49886.78	5.31	nucl	chr7:26032600..26035783 reverse
Medtr7g080170	*MtPP2C81*	502	55957.88	5.80	chlo	chr7:30476473..30479559 forward
Medtr7g081020	*MtPP2C82*	387	42953.65	8.97	nucl	chr7:30892633..30894709 forward
Medtr7g090530	*MtPP2C83*	440	49223.49	8.85	nucl	chr7:35628987..35630695 reverse
Medtr7g090540	*MtPP2C84*	271	30518.97	9.82	nucl	chr7:35632158..35633285 reverse
Medtr7g090550	*MtPP2C85*	438	49414.62	6.69	cyto	chr7:35635422..35637149 reverse
Medtr7g093240	*MtPP2C86*	129	14742.86	9.40	mito	chr7:37040877..37041934 forward
Medtr7g100240	*MtPP2C87*	370	41248.73	8.53	nucl	chr7:40325079..40328089 reverse
Medtr7g112430	*MtPP2C88*	428	46356.15	7.97	chlo	chr7:46222163..46225753 forward
Medtr7g112490	*MtPP2C89*	395	44396.30	7.17	mito	chr7:46255834..46259283 reverse
Medtr8g017240	*MtPP2C90*	373	41209.76	8.51	cyto	chr8:5799043..5800524 reverse
Medtr8g074930	*MtPP2C91*	392	43542.87	8.16	chlo	chr8:31676759..31682907 reverse
Medtr8g102550	*MtPP2C92*	402	43717.00	4.90	nucl,cyto	chr8:43176042..43184129 reverse
Medtr8g463130	*MtPP2C93*	282	30775.81	5.90	cyto	chr8:22192735..22196250 forward
Medtr0015s0140	*MtPP2C94*	387	43570.61	6.95	chlo	scaffold0015:69714..74393 reverse

To further understand the relationship between *MtPP2C* genes and *AtPP2C* genes, we further annotated the Arabidopsis homologous genes of each *MtPP2C* by Blast search against TAIR (http://www.arabidopsis.org/index.jsp) (Supplementary Table S1).

### Chromosomal location and duplication of MtPP2C genes

**Figure 1:**
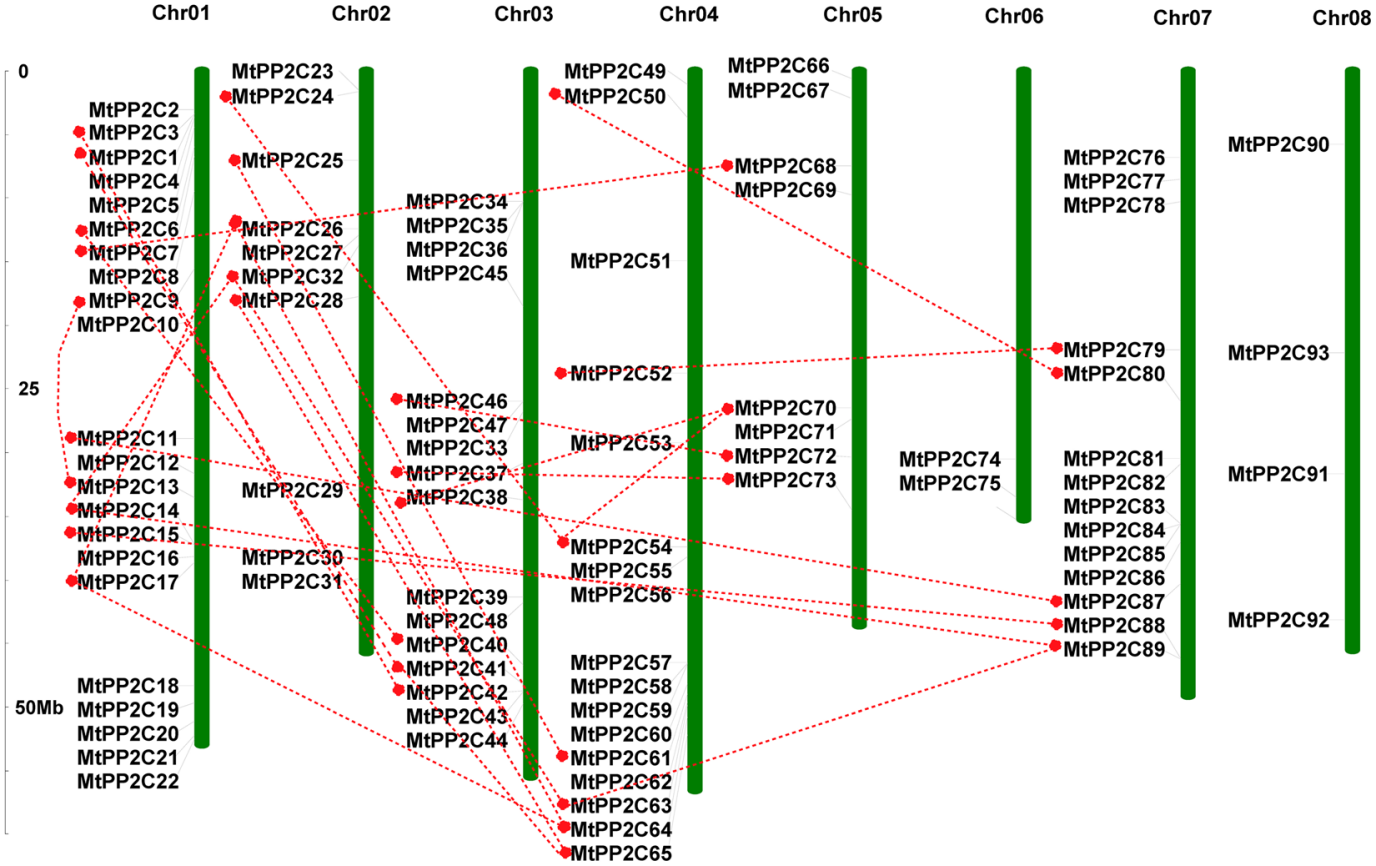
Chromosomal distribution and expansion analysis of *MtPP2C* genes in *M*. *truncatula*. Red lines show duplications between 94 *MtPP2C* genes.

Based on physical locations on *M*. *truncatula* chromosomes, the 94 *MtPP2C* genes were displayed using the MapInspect software. Ninety-three MtPP2C genes are distributed across all eight chromosomes (Ch1–Ch8), ranging from two to 22 per chromosome (Fig. 1). The number of *MtPP2Cs* located on each chromosome varies dramatically; chromosomes 1 contains the largest number of *MtPP2C* family members with 22 genes, whereas the least number was detected on chromosomes 6, containing only two *MtPP2C* genes. Furthermore, one *MtPP2C* (*MtPP2C94*) is located on an unassembled genomic scaffold, thus cannot be mapped to any particular chromosome according to what we currently know about this genome. These results showed that the *MtPP2C* genes are unevenly distributed on different chromosomes, and that each subfamily gene is also unevenly distributed.

**Figure 2:**
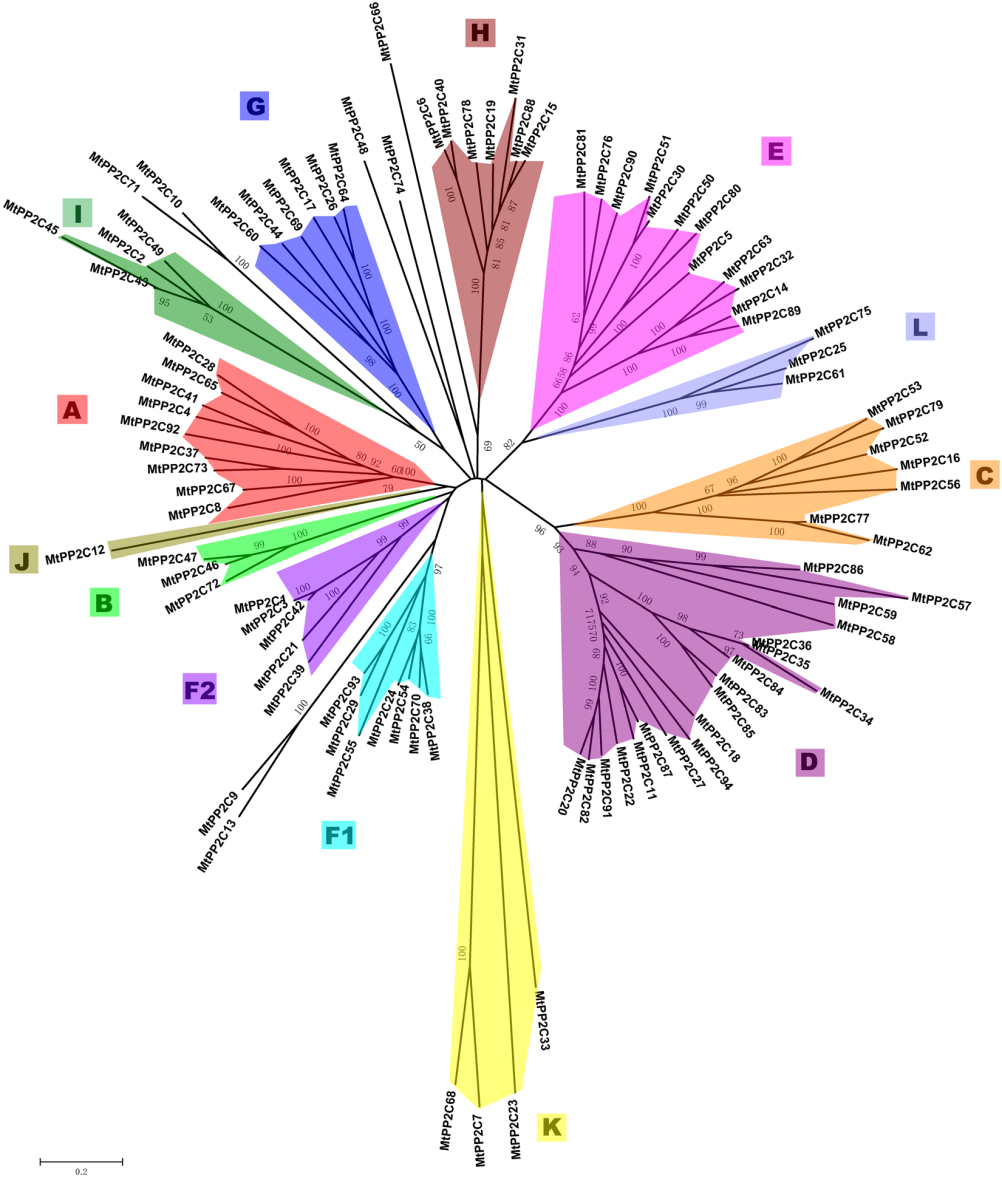
Phylogenetic relationships among 94 *MtPP2C* genes in *M*. *truncatula*. The unrooted phylogenetic tree was generated with MEGA 6.06 program using the full-length amino acid sequences of the 94 MtPP2C proteins by the neighbor-joining (NJ) method with 1,000 bootstrap replicates. Thirteen different subfamilies (A–L) are highlighted with different colored backgrounds.

Previous studies in rice, Arabidopsis and *B*. *distachyon* showed that *PP2C* gene families mainly expanded through whole-genome and chromosomal segment duplications^4,22^. Closely related genes located within a distance of less than 200 kb on the same chromosome are defined as tandem duplications, otherwise they are segmental duplications^27^. In *M*. *truncatula*, 25 pairs of paralogous *MtPP2C* genes were found to be involved in segmental duplication events and no tandem duplication gene pairs were found (Fig. 1). As shown in Fig. 1, these 25 pairs of duplicated *MtPP2C* genes are distributed on chromosome1, 2, 3, 4, 5 and 7, but not on chromosome 6 and 8. The ratio of Ka/Ks showed that 24 pairs of duplicated *MtPP2C* genes, except for *MtPP2C17/26*, have evolved mainly from purifying selection (Supplementary Table S2). Amino acid alignment and phylogenetic analysis indicated that two counterparts of each gene pair are from the same subgroup (Fig. 2 and Supplementary Table S2).

### Phylogenetic analysis

To evaluate the evolutionary relationships of 94 PP2C proteins in *M*. *truncatula*, we conducted a phylogenetic analysis using MEGA6.06 based on full-length protein sequences (Fig. 2). At the same time, we constructed another phylogenetic tree to compare the phylogenetic relationships of PP2Cs among Arabidopsis, rice and *M*. *truncatula* (Supplementary Fig. S1). Consistent with the previous studies in Arabidopsis and rice^22^, all *MtPP2C* genes are grouped into 13 subfamilies and several independent single branches. As expected, most *MtPP2Cs* cluster together with those from Arabidopsis because both *M*. *truncatula* and Arabidopsis are dicotyledonous plants, while those *PP2Cs* from rice tend to form independent branches. As shown in Fig. 2 and Supplementary Fig. S1, there is only a little difference between the two phylogenetic trees and most of the MtPP2C proteins fall into the same subfamily. In Fig. 2, MtPP2C66 can be grouped into subfamily H, while MtPP2C71 and MtPP2C10 can be grouped into subfamily I because of relatively high bootstrap support (66% and 50%, respectively), but in Supplementary Figure S1 they cannot be grouped.

As shown in Fig. 2, 87 out of 94 *MtPP2C* genes are distributed in 13 subfamilies (A-L), and the remaining seven *MtPP2C* genes, *MtPP2C9*, *MtPP2C10*, *MtPP2C13*, *MtPP2C48*, *MtPP2C66*, *MtPP2C71 and MtPP2C74*, cannot be grouped into any subfamilies. The subfamilies D, E and A are the largest three subfamilies, containing 19, 12 and 9 members, respectively. Subfamily J is the smallest one, including only one gene, *MtPP2C12*. Moreover, subfamilies C and D as well as subfamilies L and H constitute sister clades in a monophyletic cluster with high bootstrap support (96% and 86%, respectively), suggesting close evolutionary relationships between the respective subfamilies.

As shown in Supplementary Fig. 1, the number of *MtPP2C* genes in each subfamily is similar among *M*. *truncatula*, Arabidopsis and rice except for subfamily D. We found that the number of subfamily D genes in *M*. *truncatula* (19) is significantly higher than that of other plants, such as Arabidopsis (9), rice (11), maize (13) and *B*. *distachyon* (9)^4,6,22,26^. *MtPP2C57*, *MtPP2C58*, *MtPP2C59* and *MtPP2C86* are grouped into an independent branch, of which no PP2Cs from Arabidopsis and rice exist (bootstrap, 88%). Similarly, the other six genes, *MtPP2C34*, *MtPP2C35*, *MtPP2C36*, *MtPP2C83*, *MtPP2C84* and *MtPP2C85* also form an independent branch (bootstrap, 89%). These *MtPP2C* genes belonging to independent branches may have specific functions in *M*. *truncatula*. The remaining six *MtPP2C* genes from *M*. *truncatula* are clustered together with the *PP2C* genes from Arabidopsis and rice.

### Gene structure and conserved motifs distribution analysis

**Figure 3:**
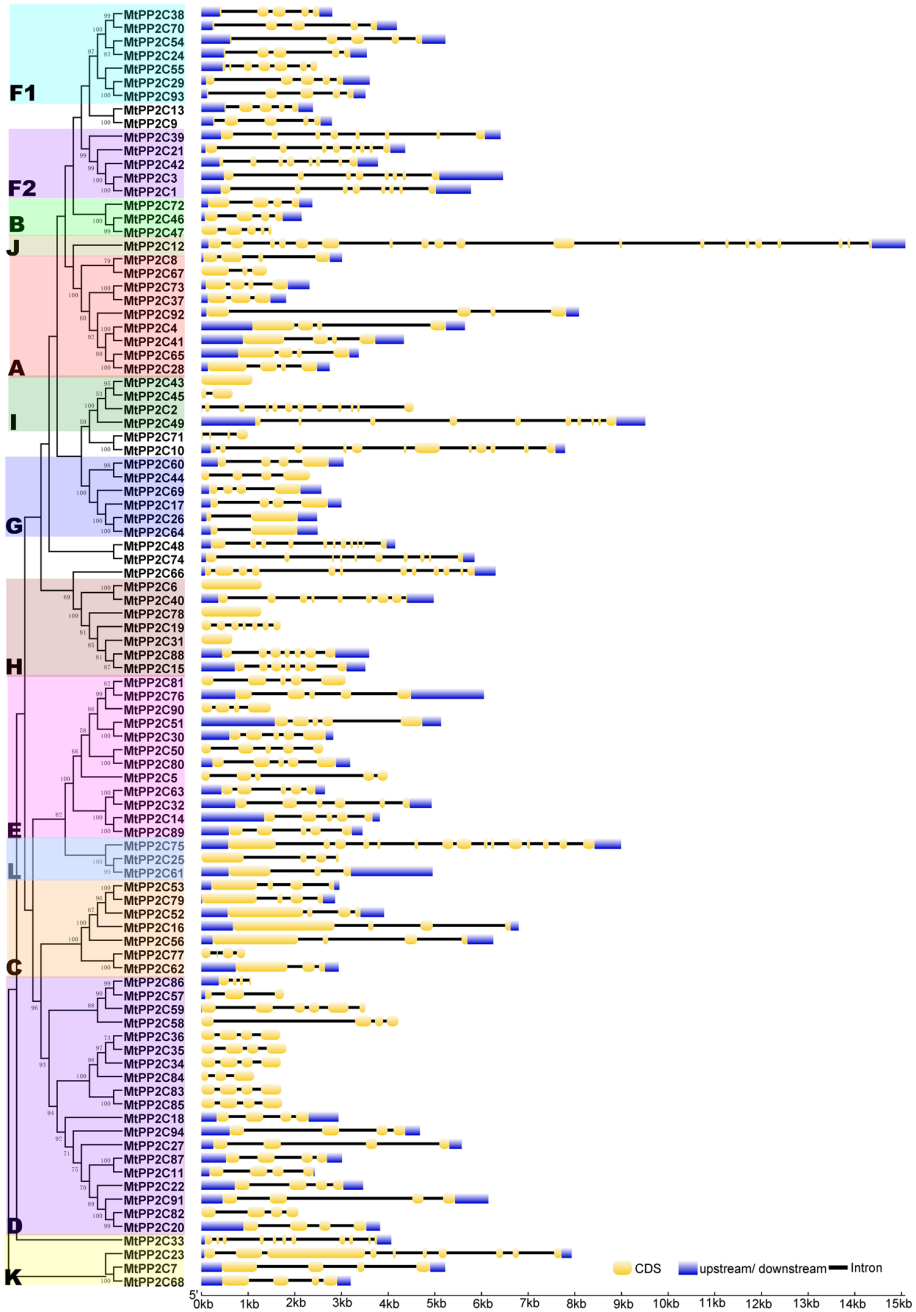
The exon-intron structure of *MtPP2C* genes. Exon-intron analyses of *MtPP2C* genes were carried out with GSDS. Lengths of exons and introns of each *MtPP2C* gene are exhibited proportionally. Gene families are grouped and color-coded based on the phylogenetic tree. For all genes, black lines represent introns, yellow boxes represent exons and purple boxes represent UTRs.

In order to better understand the conservation and diversity of motif compositions and gene structures of *MtPP2Cs*, the conserved motifs and exon-intron organization of *MtPP2Cs* were analyzed. By comparing the CDS and the genomic DNA, the *MtPP2C* gene structures were obtained (Fig. 3). The number of introns is highly divergent, from zero to 19, which is consistent with *PP2C* genes in Arabidopsis and rice. Of the 94 *MtPP2C* genes, only four genes (*MtPP2C6*, *MtPP2C31*, *MtPP2C43* and *MtPP2C78*) have no introns, whereas *MtPP2C12* contains 19 introns. In the same subfamily, most members share similar exon/intron structures, such as intron phase, intron number and exon length (Fig. 3). For example, in the largest subfamily D, 16 *MtPP2C* genes harbor three introns, with the exception of *MtPP2C57* and *MtPP2C84*, which have two introns, and *MtPP2C59*, which has five introns. In subfamily F2, all five members have seven introns. A great degree of variation in the number of introns exists in subfamilies I, H, E and K.

**Figure 4:**
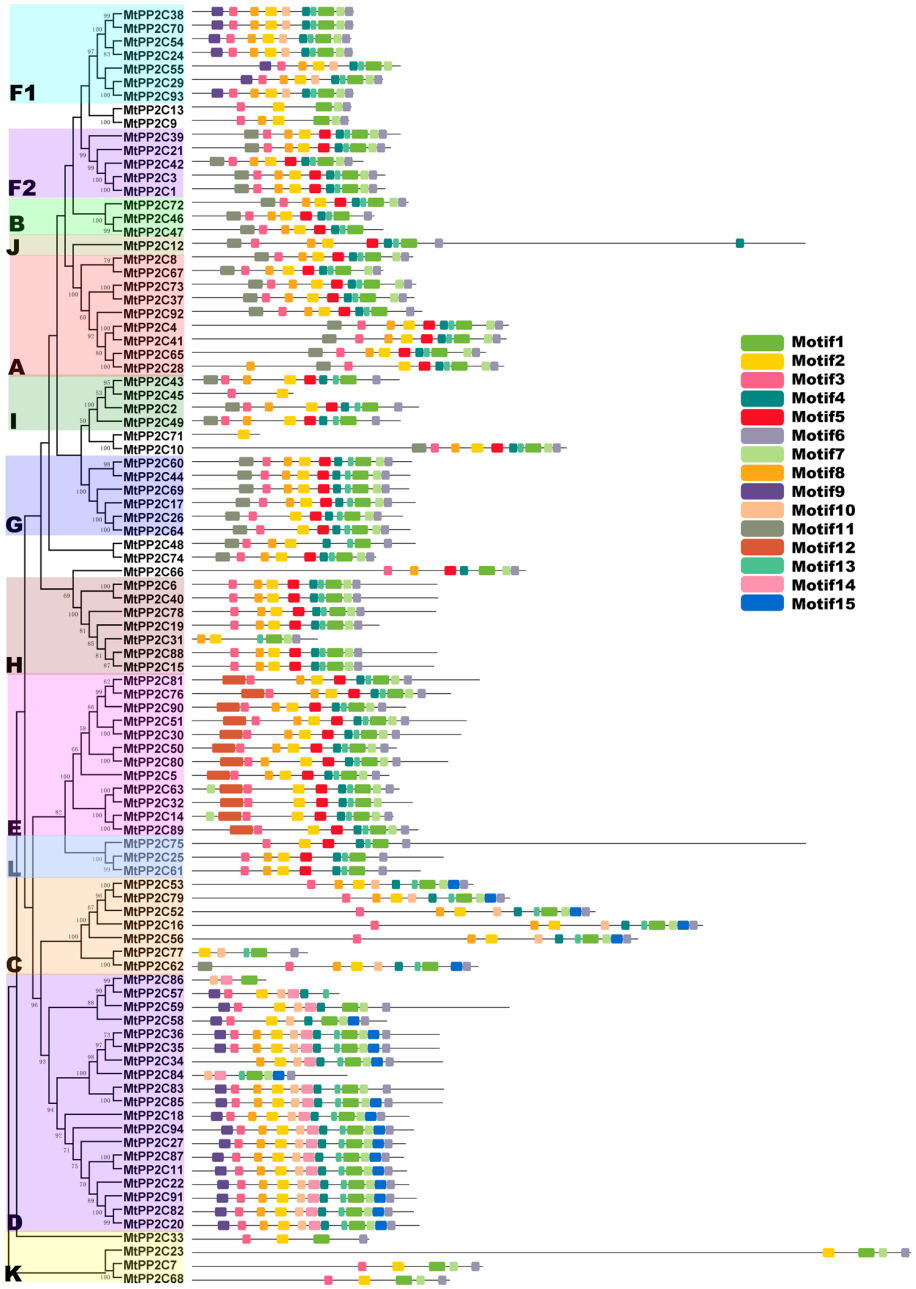
Conserved motifs of MtPP2C proteins. The conserved motifs in MtPP2C proteins were identified by MEME software. Grey lines represent the non-conserved sequences, and each motif is indicated by a colored box numbered on the right side of the figure. The length of motifs in each protein is presented proportionally.

The conserved motifs of MtPP2C proteins were analyzed using the software MEME, and 15 distinct conserved motifs were identified (Supplementary Fig. S2). The composition patterns of motifs tend to be consistent with the results from our phylogenetic tree, that is to say, the MtPP2Cs within each subfamily share similar motif compositions, but among different subfamilies, the motif compositions vary (Fig. 4). Motif 1, 2, 3, 4, 6, 7, 8 and 13 are present in most subfamilies, among them, motif 2 is present in 91 MtPP2C proteins except for MtPP2C66, MtPP2C86 and MtPP2C84. In contrast, some other motifs exist only in specific subfamilies. For instance, motif 12 and motif 14 is present only in subfamilies E and D, respectively, while motif 9 is present in both subfamilies F1 and D. These results suggest that the specific functions of different subfamily genes may be due to specific motifs. This indicates that patterns of introns and motifs, which correlate well with the phylogenetic clades, strongly support their close evolutionary relationships among the *MtPP2C* genes within the same subfamilies.

### Cis-element analysis in the promoter regions of MtPP2Cs

Cis-elements in combination with transcription factors regulate the transcription level of a gene. To investigate the possible roles of *MtPP2Cs* in abiotic stresses, corresponding promoter regions (1.5 kb upstream ATG) of 94 *MtPP2C* genes was subjected to cis-element analysis by PlantCARE online.

Fourteen putative cis-acting elements were investigated in this study (Supplementary Table S3), including six abiotic stress-responsive (ARE, C-repeat/DRE, HSE, LTR, MBS and TC-rich repeats) and nine hormone-responsive (ABRE, CGTCA-motif, ERE, GARE-motif, P-box, TATC-motif, TCA element and TGA-element) cis-acting elements. Overall, cis-elements responsive to abiotic stresses and hormones are widely present in the promoters of the *MtPP2C* genes and the number of cis-elements ranges from 3 to 18 (Supplementary Table S4), suggesting that these *MtPP2Cs* are involved in responses to different stresses in *M*. *truncatula*.

### Expression Profiles of the *MtPP2C* Genes in Different Tissues

Sixteen *MtPP2C* genes (*MtPP2C9*, *13*, *16*, *25*, *31*, *45*, *46*, *50*, *55*, *56*, *57*, *67*, *77*, *78*, *79* and 86) do not have their corresponding probe sets in the dataset, but the expression profiles of the rest 78 *MtPP2C* genes were analyzed (Supplementary Fig. S3). Different *MtPP2C* genes show different expression patterns in each tissue. Some genes are highly expressed in all eight tissues, such as *MtPP2C20*, *MtPP2C29*, *MtPP2C39*, *MtPP2C73*, *MtPP2C91* and *MtPP2C93*. In contrast, the expression of some genes is low in all eight tissues, such as *MtPP2C34*, *MtPP2C35* and *MtPP2C36*. Some *MtPP2C* genes show significantly distinct tissue-specific expression patterns across the eight tissues examined. For instance, *MtPP2C32* is preferentially expressed in roots but lowly expressed in other seven tissues. In another example, the expression of *MtPP2C11* in roots and nodules is much lower than that in the other six tissues, but the expression of *MtPP2C5* is exactly the opposite of *MtPP2C11*. The results revealed that different *PP2C* genes from *M*. *truncatula* might function in different tissues.

### Expression Profiles of *MtPP2C* Genes Under Cold, Drought and ABA Stress

In plants, many *PP2Cs* play important roles in response to drought and cold stresses. To investigate the expression profiles of *MtPP2C* genes under different abiotic stress, quantitative real time-PCR (qRT-PCR) analysis was used to examine their transcription levels.

**Figure 5:**
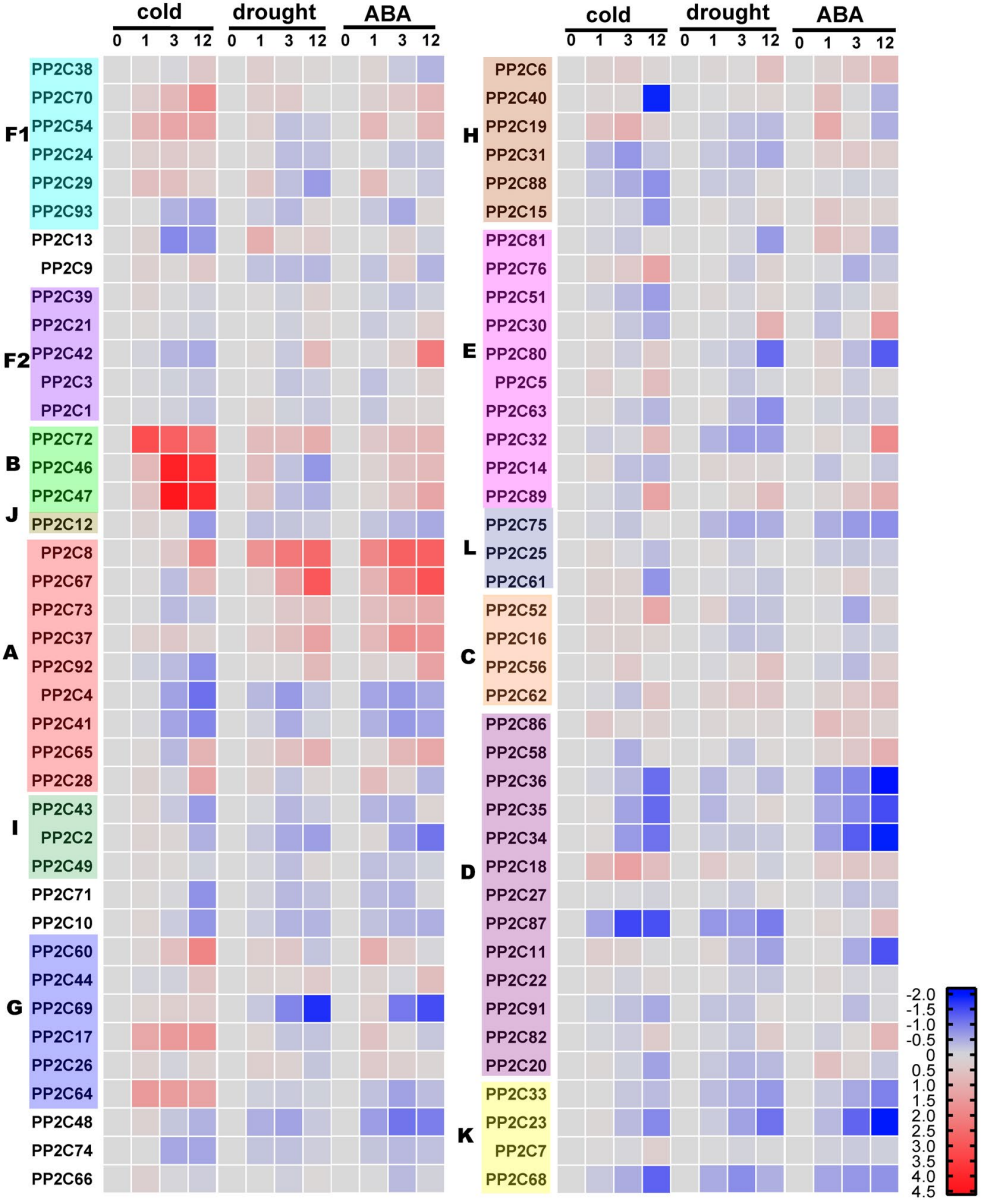
Relative transcriptional expression levels of MtPP2Cs under different abiotic treatments. Each column indicates a sampling time point, and each row indicates an MtPP2C member. The expression level of the control (at 0 h; marked in gray) in every treatment for each gene is used as the rescaled value when calculating the relative expression levels. The relative expressions are log2 transformed and visualized for heatmap using Graphpad prism 7. The colors vary from blue to red representing the scale of the relative expression levels.

In our study, transcripts of 80 *MtPP2C* genes could be detected by qRT-PCR (CT vaule ≤ 35), but transcripts of 14 *MtPP2C* genes was barely detectable (*MtPP2C45*, *MtPP2C50*, *MtPP2C53*, *MtPP2C55*, *MtPP2C57*, *MtPP2C59*, *MtPP2C77*, *MtPP2C78*, *MtPP2C79*, *MtPP2C83*, *MtPP2C84*, *MtPP2C85*, *MtPP2C90* and *MtPP2C94*). As shown in Fig. 5, we found that many *MtPP2C* genes tested in this study show similar trends under three different treatments, especially under drought and ABA treatments. On the contrary, some genes have different expression patterns under different treatments. Furthermore, the *MtPP2C* genes with significantly altered expression after treatments (fold change ≥ 2 than controls in all three independent treatments) were selected and listed in Supplementary Table 5.

All together, we obtained 24 *MtPP2C* genes showing significant differences in expression levels under cold stress, including 14 up-regulated and 10 down-regulated genes. Three genes belonging to subfamily B, *MtPP2C72*, *MtPP2C46* and *MtPP2C47*, were most significantly up-regulated under cold treatment, implying their important roles in the response to cold stress. The expression levels of five genes belonging to subfamily D changed significantly under cold treatment, four (*MtPP2C34*, *MtPP2C35*, *MtPP2C36* and *MtPP2C87*) of which were down-regulated and one (*MtPP2C18*) was up-regulated. Similarly, the expression levels of the four genes belonging to subfamily A changed remarkably, three (*MtPP2C4*, *MtPP2C41* and *MtPP2C92*) of which were down-regulated and one (*MtPP2C8*) was up-regulated. In addition, some *MtPP2C* genes from other subfamilies were also induced or inhibited by cold treatment.

Under drought treatment, 11 *MtPP2C* genes showed obviously different expression levels, including six up-regulated and five down-regulated genes. The expression levels of five genes belonging to subfamily A, *MtPP2C8*, *MtPP2C37*, *MtPP2C65*, *MtPP2C67* and *MtPP2C92*, were all up-regulated obviously, and another subfamily A *MtPP2C* genes, *MtPP2C73*, was also up-regulated but at a lower degree (fold change ≥ 1.5). The expression level of *MtPP2C69*, which belongs to subfamily G, was the most obviously down-regulated under drought treatment.

Under ABA treatment, 14 *MtPP2C* genes exhibited different expression levels, including nine up-regulated and five down-regulated genes. The *MtPP2C* genes with increased expression levels after ABA treatment are highly correlated with those responsive to drought treatment, such as *MtPP2C8*, *MtPP2C37*, *MtPP2C65*, *MtPP2C67*, *MtPP2C67*, *MtPP2C92* and *MtPP2C30*.

Among the *MtPP2C* genes with significantly altered expression levels after different treatments, *MtPP2C8* is the only gene that was up-regulated by all three treatments. Unlike *MtPP2C8*, the expression level of *MtPPC92* was increased significantly by drought and ABA treatments, while decreased significantly by cold treatment. The expression levels of some *MtPP2C* genes changed significantly by two treatments, such as *MtPP2C67*, *MtPP2C73*, *MtPP2C 37*, *MtPP2C23*, *MtPP2C69* and *MtPP2C80* under drought and ABA treatment, and *MtPP2C34*, *MtPP2C35* and *MtPP2C36* under drought and cold treatment. In addition, the expression level of some genes changed only by one treatment, such as *MtPP2C40* by cold treatment. Different expression patterns of *MtPP2C* genes may indicate different roles in response to different treatments.

## Discussion

Based on the completion of *M*. *truncatula* genome sequencing^28^, many gene families were identified and characterized at the whole-genome level, including CCCH^29^, LBD^30^, WRKY^31^, AP2/ERF^32^, Dof ^33^, GH3^34^, CAMTA^35^, LEA^36^, MAPKKK^37^, U-box^38^, MYB^39,40^ and GRAS^41,42^. In this study, *PP2C* genes in *M*. *truncatula* were comprehensively studied, from genome-wide identification, chromosomal locations, evolutionary relationships, gene structure and conserved motifs analysis to expression patterns under cold and drought stresses.

Compared to other gene families, the *PP2C* gene family is one of the largest families in the plant kingdom. Genome-wide analyses have identified 80, 90, 91, 88, 104 and 86 *PP2C* gene family members in Arabidopsis^22^, rice^6^, tomato, hot pepper^24^, maize^26^, and *B*. *distachyon*^4^ genomes, respectively. Evolutionary analysis showed that *PP2C* genes are divided into 11,12 or 13 groups in different plants. In lower plants, such as *Chlamydomonas reinhardtii*, *Physcomitrella patens* and *Selaginella tamariscina*, the *PP2C* gene family members are much less than those in higher plants. The increase and expansion of *PP2C* genes from lower plants to higher plants may correlate with adaptations to complex environmental conditions^43^. Here, we identified 94 *MtPP2C* genes from *M*. *truncatula* and divided them into 13 subfamilies (Table 1 and Fig. 1), consistent with other higher plants, such as tomato and hot pepper^24^.

Most proteins in the same MtPP2C subfamily share similar parameters and the number of introns except for subfamily K. Different subfamilies of MtPP2Cs are distinguished from each other in the values of MW and pI (Table 1 and Supplementary Fig. S4). Overall, members of most subfamilies have a more concentrated MW distribution (30–60) and a wider pI distribution (4.5–10). In contrast, members in subfamilies C and L have a wider MW distribution (22.6–119.58) and a concentrated pI distribution (4.94–6.64). Neither MW nor PI distribution is concentrated in the members of subfamilies K and J (Table 1 and Supplementary Fig. S4). Similar to MW, pI, and the number of introns, MtPP2C proteins grouped into the same subfamily exhibit similar motif distributions, suggesting functional similarities for members in the same subfamily.

Subfamilies A and B *PP2C* genes only exist in plants. Members of subfamily A play a role in ABA-dependent stress responses, while members of subfamily B have been characterized as regulators of MAPK activities^43^. In this study, expression pattern analysis showed that subfamilies A and B exhibit the most prominent responses to abiotic stresses among all 13 MtPP2C subfamilies (Fig. 5 and Supplementary Table S5).

Studies on model organisms Arabidopsis and rice demonstrated that family A PP2C plays an important role in plant response to abiotic stress, especially in the ABA signaling pathway^2,44^. After evolutionary analysis and sequence alignment, nine *PP2C* genes belonging to family A in *M*. *truncatula* were identified. Consistent with reports in other plants, most members in subfamily A in *M*. *truncatula* are significantly up- or down-regulated under cold and drought stress. Furthermore, those subfamily A genes significantly up-regulated by drought are induced by ABA as well, indicating that they are regulated by ABA-dependent pathways.

*MtPP2C8*, *MtPP2C37*, *MtPP2C67* and *MtPP2C73*, which are homologs of *HAI* PP2Cs (Highly ABA-Induced1,2,3) in Arabidopsis, are significantly induced by drought and ABA treatment, while *MtPP2C8* is also significantly induced by cold treatment (Fig. 5 and Supplementary Table 5). Studies in Arabidopsis have shown that *HAI* PP2Cs have unique drought resistance functions. *HAI* PP2Cs have the greatest effect on ABA-independent low water potential phenotypes but have lesser effect on classical ABA sensitivity phenotypes^44^.

The expression of *MtPP2C92* and *MtPP2C65* is increased significantly under drought and ABA treatment, but the expression of *MtPP2C92* is decreased under cold treatment (Fig. 5 and Supplementary Table S5). In Arabidopsis, *ABI1* (homolog of *MtPP2C92*) and *ABI2* (homolog of *MtPP2C65*) are two most extensively studied PP2Cs and have been characterized as the main components of the ABA signaling pathway under abiotic stresses and during development^2,43,45^. The function of *MtPP2C92* and *MtPP2C65* in *M*. *truncatula* may be similar to that of *ABIs* in Arabidopsis, but the different expression patterns after cold treatment may indicate their differences in cold responses.

There are six members of subfamily B PP2Cs in Arabidopsis^22^, four of them (*AP2C1–4*) maintain a kinase interaction motif at the N-terminal region of the proteins and are characterized as MAPK phosphatases^3^. Only three members of subfamily B PP2C (*PP2C46*, *PP2C47* and *PP2C72*) in *M*. *truncatula* were identified. Phylogenetic analysis indicates that they are closely related to *AP2C1-4* (Supplementary Fig. S1 and Supplementary Table S1). AP2C1, a homolog in Arabidopsis with MtPP2C46 and MtPP2C47, was reported as a negative regulator of stress-induced MAP kinase cascade by interacting with and inactivating Arabidopsis MPK4 and MPK6. *AP2C1* modulates innate immunity and stress hormones such as jasmonic acid and ethylene in Arabidopsis^46^. In alfalfa, MP2C (homolog with AP2C1) functions as a negative regulator of the stress-activated MAPK pathway that is activated by cold, drought, touch, and wounding^47^. AP2C2, a homolog in Arabidopsis with MtPP2C72, is a regulator of stress response signaling, in particular ROS signaling activated by both biotic and abiotic stresses^48^. Expression analysis showed that the expression of *MtPP2C46*, *MtPP2C47* and *MtPP2C72* is induced by cold, drought and ABA, especially by cold treatment (Fig. 5 and Supplementary Table S5). In Arabidopsis, *AP2C1* expression is strongly induced by cold, drought and wounding, but *AP2C2* is slightly induced by these treatments^48^. The above studies indicate that subfamily B *PP2C* genes in *M*. *truncatula* may be regulators of the stress-induced MAP kinase cascade, similar to those in Arabidopsis, but the specific function may be different. In *M*. *truncatula*, *MtPP2C46*, *MtPP2C47* and *MtPP2C72* may play a vital role in cold responses.

In addition to the *PP2C* genes from subfamilies A and B, many *PP2C* genes from other subfamilies have also been reported to respond to abiotic stress in plants. Similar to reports in other plants, our study in *M*. *truncatula* also revealed that some *MtPP2Cs* from other subfamilies are induced by cold and drought. The expression of several genes in subfamily E is significantly altered after treatments, such as *MtPP2C89* under cold and ABA treatments (Fig. 5 and Supplementary Table S5). A recent study showed three *EGRs* (Clade E Growth-Regulating) (homolog of *MtPP2C89*), which belong to subfamily E PP2C in Arabidopsis, act as negative growth regulators to restrain growth during drought^49^. However, the function of other subfamily PP2C in plant resistance to abiotic stress is poorly understood and needs to be further investigated.

The results of our study establish a foundation for future studies on the functions of *MtPP2C* genes in plant abiotic response, and provide a basic understanding that may allow us to elucidate the potential functions of *MtPP2C* genes under drought and cold stresses in *M*. *truncatula*.

## Methods

### Database Searches and Identification of *PP2C* Genes in *M*. *truncatula*

The InterPro PP2C domain “IPR001932” was used to search the Plaza3.0 database (http://bioinformatics.psb.ugent.be/plaza/) in order to identify PP2C candidate genes in *M*. *truncatula*^50^. Amino acid sequences (Supplementary Data 1), CDS sequences (Supplementary Data 2**)** and Genomic sequences (Supplementary Data 3) of *PP2C* genes in *M*. *truncatula* were downloaded from the Phytozome12.1 database (https://phytozome.jgi.doe.gov/pz/portal.html)^51^. All protein sequences were manually checked individually using Pfam (http://pfam.xfam.org/) and the online Batch CD-search (https://www.ncbi.nlm.nih.gov/Structure/bwrpsb/bwrpsb.cgi) to confirm the presence of the PP2C domains^52,53^. All candidate *PP2C* genes with no PP2C domains were removed.

Proteins of PP2Cs in Arabidopsis and rice were downloaded from the TAIR database (https://www.arabidopsis.org) and the Rice Genome Annotation Project Database (https://rice.plantbiology.msu.edu/), which was described in previous reports^4,22^.

### Analysis of protein features and chromosomal locations

The Compute pI/MW tool of the ExPASy server (http://web.expasy.org/compute) was used to calculate the molecular weight (MW) and the theoretical isoelectric point (pI) of MtPP2C proteins. The WoLF PSORT program (https://wolfpsort.hgc.jp/) was used to predict protein subcellular localization^54^.

According to the starting positions on chromosomes, the MapInspect software was used to draw the chromosomal distribution images of of *MtPP2C* genes.

Duplications between the *PP2C* genes were identified and complemented using the PGDD database (http://chibba.agtec.uga.edu/duplication/)^55,56^. The number of nonsynonymous substitutions per nonsynonymous site (Ka) and the number of synonymous substitution per synonymous site (Ks) of duplicated genes were obtained from PGDD database. Ka/Ks < 1 means purifying selection; Ka/Ks = 1 means neutral selection; while Ka/Ks > 1 means positive selection^57^.

### Phylogenetic tree, gene structure and conserved motifs

The protein sequences of *MtPP2C* genes were aligned by ClustalW^58^ and used for phylogenetic analysis using MEGA6.06^59^, and an unrooted phylogenetic tree was constructed using the neighbor-joining (NJ) method with the following parameters: Poisson correction, pair-wise deletion, and 1,000 bootstrap replicates.

The exon-intron structures of *MtPP2C* genes were determined by comparing the coding sequences and the corresponding genomic sequences on the GSDS website (http://gsds2.cbi.pku.edu.cn)^60^.

The MEME software (Version 4.11.4) was used to identify conserved motifs in MtPP2C protein sequences according to the following parameters: -protein, -oc, -nostatus, -mod zoops, -nmotifs 15, -minw 6, -maxw 50^61^.

### Cis-elements analysis

The 1,500 bp sequences upstream from the initiation codon (ATG) of all *MtPP2C* genes (Supplementary Data 4) were obtained from Phytozome v12.1^51^. The putative stress and hormone responsive cis-elements in the promoter regions were identified using the PlantCARE (http://bioinformatics.psb.ugent.be/webtools/plantcare/html/) program. The details of six abiotic stress-responsive and nine hormone-responsive cis-elements investigated in this study were list in Supplementary Table 6.

### Expression profiling of the *MtPP2C* genes in different tissues

The expression profile of *MtPP2C* genes in eight tissues (root, stem, leaf, vegetative bud, petiole, flower, pod and nodule) were analyzed using *M*. *truncatula* microarray data^62^.

The genome-wide microarray data were obtained from the *M*. *truncatula* Gene Expression Atlas (MtGEA) Project website (http://mtgea.noble.org/v2/). The relative expressions were log2 transformed and visualized for heat map using Graphpad prism 7.

### Plant materials, growth conditions and abiotic stress treatments

*M*. *truncatula* ecotype Jemalong A17 was used in this study. The seeds were first treated with sulfuric acid and washed with sterilized water, then sown in a mixture of peat soil and vermiculite (1:1, V/V). Seedlings were grown at 22–24 °C in a growth chamber with a 16/8 h (day/night) photoperiod until they were used for treatment at eight weeks old. The method of stress treatment is in accordance with Shu’s report^29^. For cold stress treatment, the seedlings were transferred to the 4 °C incubator. For drought stress treatment, the seedlings growing under normal conditions were watered with 300 mM mannitol solution. For ABA treatment, the seedling leaves were sprayed with 100 μM ABA solution. The seedlings were harvested at 0, 1, 3 and 12 hours after treatment. For each treatment, five randomly chosen whole seedlings were pooled to form a biological replicate. All samples were frozen immediately in liquid nitrogen after harvest and stored at −80 °C until used for RNA extraction.

### Expression analysis of *MtPP2C* genes response to abiotic stress

Total RNA was isolated from all of the samples using the total RNA extraction kit (Tiangen, China). The quality and quantity of RNA was evaluated by agarose gel electrophoresis and Quawell micro volume spectrophotometer (Q5000, USA), respectively. Then, 1 µg of total RNA after DNase I digestion was reverse transcribed into cDNA using the PrimeScript™ II 1st Strand cDNA Synthesis Kit (TaKaRa, Japan).

The cDNA was amplified using LightCycler 480 SYBR Green Master, with a Roche LightCycler 480 Real Time PCR system (Roche, Switzerland). The thermal cycling program was 95 °C for 30 s, followed by 40 cycles of 95 °C for 5 s, 60 °C for 30 s and 72 °C for 15 s. The melting curves were analyzed at 60–95 °C after 40 cycles. All qRT-PCRs were carried out for three technical replicates. The relative expression levels of *MtPP2C* genes were calculated according to the method of Livak and Schmittgen^63^. *MtActin* (*Medtr2g008050*) and *MtGapdh* (*Medtr3g085850*) were used as reference genes. The primers used in this study were listed in Supplementary Table S6. The relative expressions were log2 transformed and visualized for heat map using Graphpad prism 7.

## Electronic supplementary material

Supplementary Figures

Supplementary Tables

Dataset
