# Supplementary material for: Genome-wide Identification of PP2C Genes and Their Expression Profiling in Response to Drought and Cold Stresses in Medicago truncatula
Source: Sci Rep. 2018 Aug 27;8:12841. doi: 10.1038/s41598-018-29627-9 (PMC6110720; doi:10.1038/s41598-018-29627-9)
Supplement: Supplementary file 1 — Supplementary Figures [file 41598_2018_29627_MOESM1_ESM.pdf]

Genome-wide Identification of PP2C Genes and Their Expression Profiling in Response to Drought and Cold Stresses in *Medicago truncatula*

Qi Yang[1#], Kun Liu[1#], Xiaocui Niu[1], Qi Wang[3], Yongqing Wan[1], Feiyun Yang[1], Guojing Li[1], Yufen Wang[2]* & Ruigang Wang[1]*

[1]Inner Mongolia Key Laboratory of Plant Stress Physiology and Molecular Biology, College of Life Sciences, Inner Mongolia Agricultural University, Hohhot, P. R. China.
[2]Key Laboratory of Forage and Endemic Crop Biotechnology, Ministry of Education, School of Life Sciences, Inner Mongolia University, Hohhot, P. R. China.
[3]Institute of Microbiology, Chinese Academy of Sciences, Beijing, P. R. China.

[#] Qi Yang and Kun Liu contributed equally to this work.

Correspondence author:

Ruigang Wang, wangruigang@imau.edu.cn; Yufen Wang, fenwy300@163.com.

Supplementary Figures

Supplementary Figure S1. Phylogenetic analysis of PP2C proteins among *M. truncatula*, Arabidopsis and rice. The phylogenetic tree was constructed with MEGA 6.06 program using the full-length amino acid sequences of the PP2C proteins by the neighbor-joining (NJ) method with 1,000 bootstrap replicates.

Supplementary Figure S2. Sequence logos for the conserved motifs of MtPP2C domain proteins.

Supplementary Figure S3. Heatmap showing the *MtPP2C* genes expression pattern in eight tissues of *M. truncatula*. The color scale at the top of each dendrogram represents log2 expression values, green represents a low level and red indicates a high level of transcript abundance.

Supplementary Figure S4. Putative MW and PI of MtPP2C proteins in *M. truncatula*. The genes in the 13 subfamilies were distinguished by different legends.

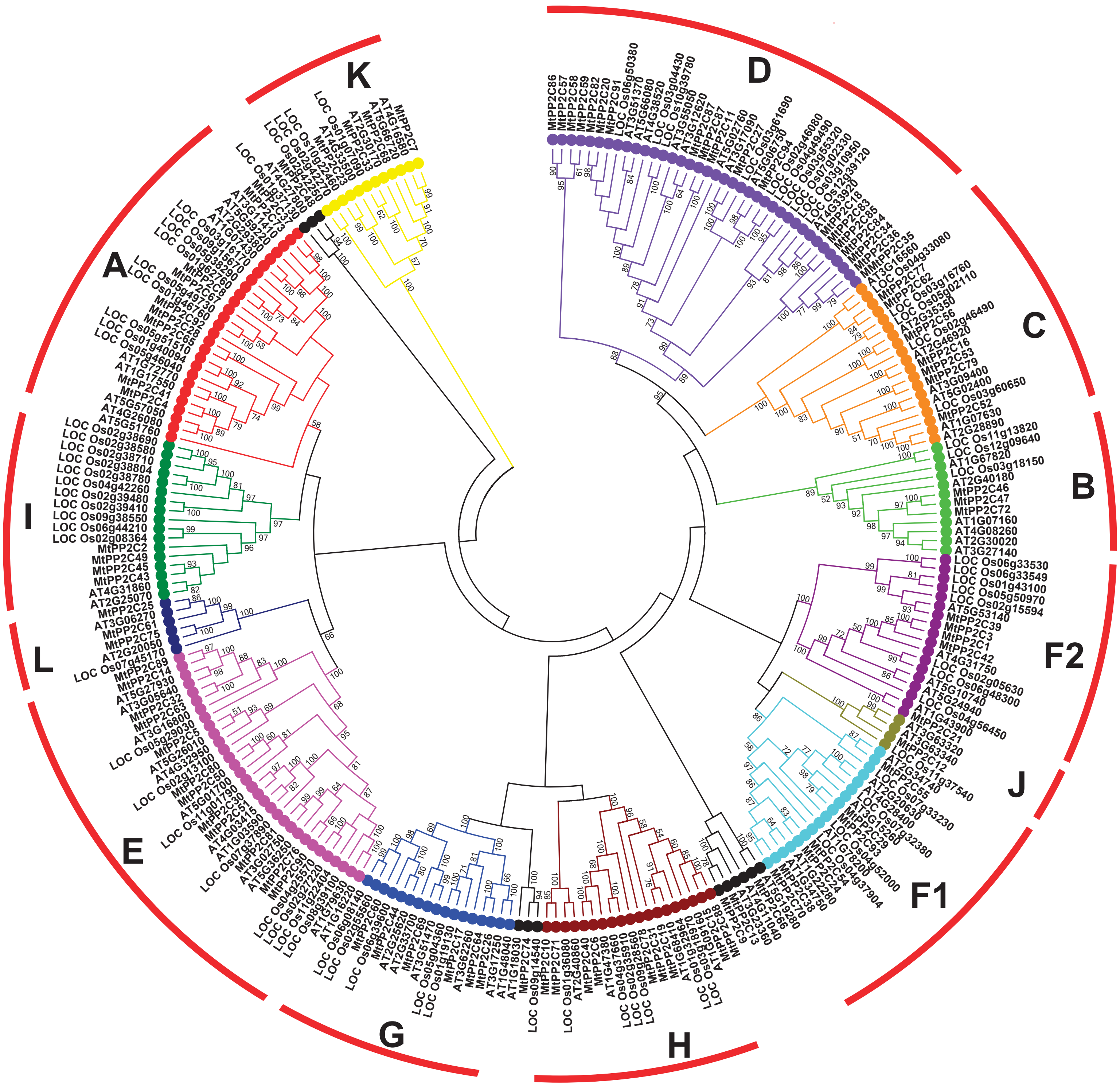

Supplementary Figure S1. Phylogenetic analysis of PP2C proteins among *M. truncatula*, Arabidopsis and rice. The phylogenetic tree was constructed with MEGA 6.06 program using the full-length amino acid sequences of the PP2C proteins by the neighbor-joining (NJ) method with 1,000 bootstrap replicates.

Motif 1
E-value: 6.0e-1146 Sites:85 Width:29

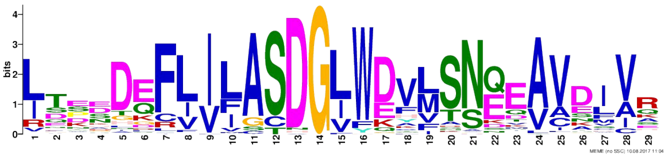

Motif 2
E-value: 5.6e-757 Sites:91 Width:21

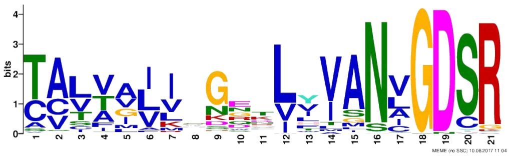

Motif 3
E-value: 1.2e-550 Sites:86 Width:15

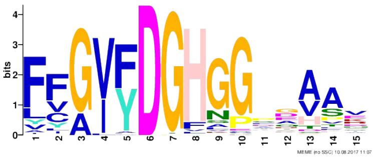

Motif 3
E-value: 1.3e-519 Sites:83 Width:15

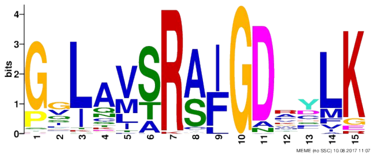

Motif 5
E-value: 1.4e-377 Sites:51 Width:21

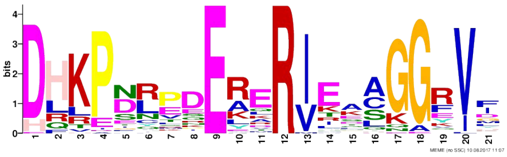

Motif 6
E-value: 3.3e-383 Sites:85 Width:15

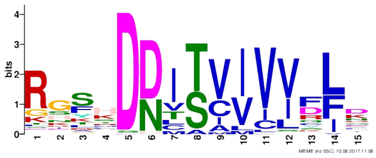

Motif 7
E-value: 2.20e-182 Sites:74 Width:15

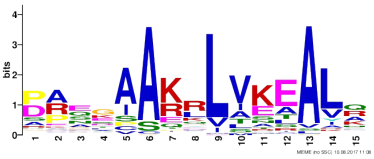

Motif 8
E-value: 1.10e-152 Sites:75 Width:15

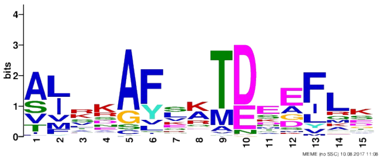

Motif 9
E-value: 1.20e-146 Sites:19 Width:21

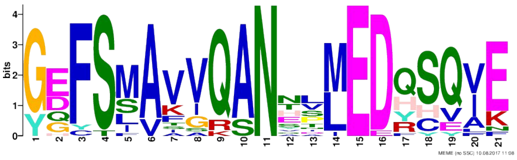

Motif 10
E-value: 3.10e-131 Sites:33 Width:15

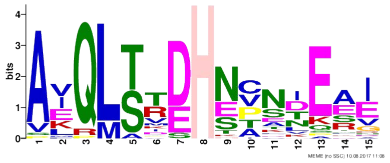

Motif 11
E-value: 2.10e-177 Sites:28 Width:26

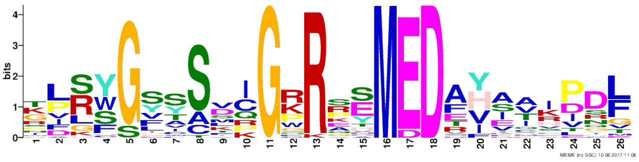

Motif 12
E-value: 4.70e-151 Sites:12 Width:41

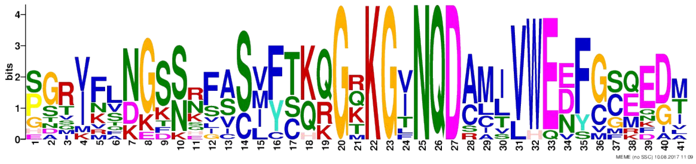

Motif 13
E-value: 1.00e-128 Sites:86 Width:11

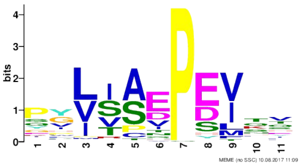

Motif 14
E-value: 5.60e-122 Sites:14 Width:21

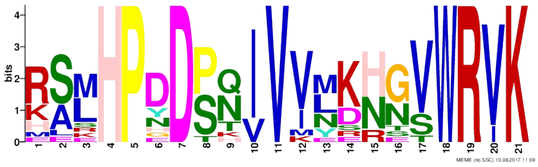

Motif 15
E-value: 1.40e-115 Sites:14 Width:21

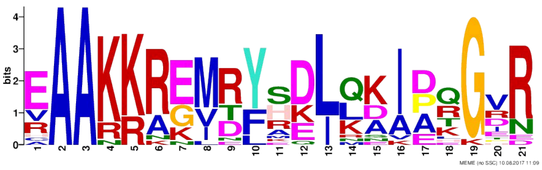

Supplementary Figure S2. Sequence logos for the conserved motifs of MtPP2C domain proteins.

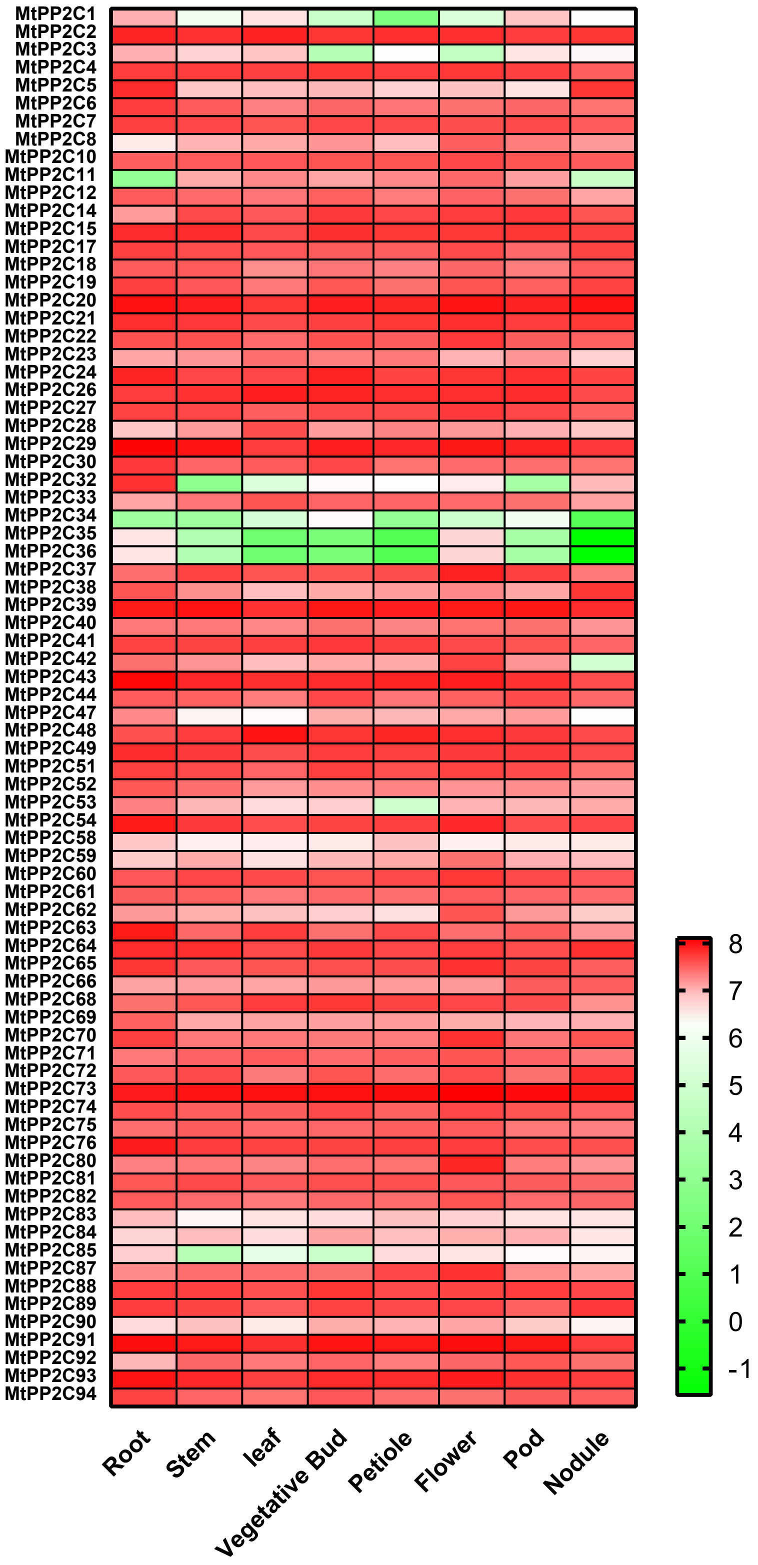

Supplementary Figure S3. Heatmap showing the *MtPP2C* genes expression pattern in eight tissues of M. truncatula. The color scale at the top of each dendrogram represents log2 expression values, green represents a low level and red indicates a high level of transcript abundance.

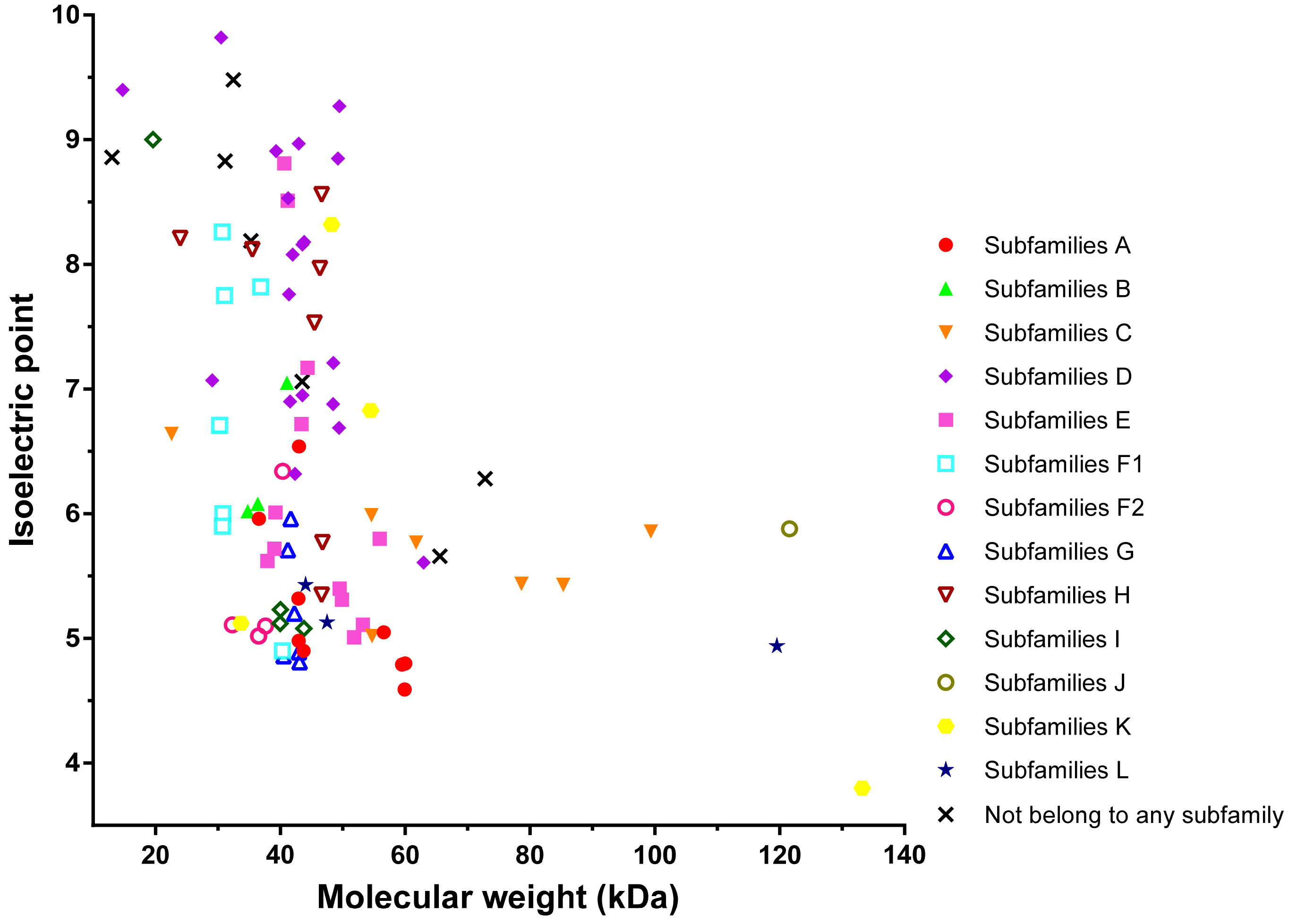

Supplementary Figure S4. Putative MW and PI of MtPP2C proteins in *M. truncatula*. The genes in the 13 subfamilies were distinguished by different legends.
